# Heat acclimation induces AcLBD1 activated AcHSFA2s mutual amplification cascade for rapid recovery of root vitality and leaf photosynthesis in kiwifruit

**DOI:** 10.3389/fpls.2026.1821100

**Published:** 2026-05-05

**Authors:** Ke-jia Zhang, Fang Li, Lu-lu Chen, Wen-yue Su, Ying-ying Wu, Xue-ren Yin, Xiao-fen Liu

**Affiliations:** 1 One Belt and One Road International Joint Research Center of Horticultural Products Quality and Post-Harvest Biotechnology in Anhui Province, School of Horticulture, Anhui Agricultural University, Hefei, Anhui, China; 2 Anhui Province Key Laboratory of Horticultural Crop Quality Biology, School of Horticulture, Anhui Agricultural University, Hefei, Anhui, China; 3 College of Agriculture and Biotechnology, Zhejiang University, Hangzhou, Zhejiang, China

**Keywords:** heat acclimation, kiwifruit, LBD−HSFA2−HSP20 cascade, leaf photosynthesis, root vitality, transformation

## Abstract

**Introduction:**

Kiwifruit (*Actinidia* spp.) is a succulent, shallow-rooted perennial fruit tree highly sensitive to environmental fluctuations, particularly the increasingly frequent extreme heat events.

**Methods:**

This study investigated heat acclimation (HA) involving a transition from mild (35 °C) to extreme (45 °C) heat stress in heat-sensitive kiwifruit, aiming to elucidate the molecular biological mechanisms underlying HA responses in perennial plants.

**Results:**

Results revealing the enhanced recovery of plants under 55 °C stress via improved root vitality and photosynthesis. Transcriptomics identified *AcLBD1* as a key acclimation-responsive gene that upregulates *AcHSFA2-1*. The *achsfa2-1* loss-of-function mutant displayed completely impaired thermotolerance, with significantly suppressed expression of *AcHSFA2-2* in its transcriptome. Overexpressing *AcHSFA2-2* increased root vitality as high as 4.13‑fold and boosted post‑heat‑stress recovery of 35S::*AcHSFA2-2* kiwifruit. Moreover, AcHSFA2‑1 and AcHSFA2‑2 form a positive feedback loop, that AcHSFA2‑1 significantly enhanced the promoter vitality of *AcHSFA2-2*, and vice versa. And the AcLBD1‑AcHSFA2s module co‑activates AcHSP20s encoding heat-protective proteins (with AcHSFA2s more potent than AcLBD1).

**Discussion:**

These findings establish a LBD‑HSFA2‑HSP20 cascade network governing heat resilience via root and photosynthetic recovery, advancing the molecular basis of plant heat acclimation.

## Highlights

- Heat acclimation enhances root vitality recovery in heat-damaged kiwifruit, which underlies the rapid restoration of leaf net photosynthetic rate and improved thermotolerance.
- *AcHSFA2–1* responds to heat acclimation; the *achsfa2–1* loss-of-function mutant completely loses thermotolerance, with strongly repressed *AcHSFA2–2* expression.
- *AcHSFA2–1* and *AcHSFA2–2* mediate mutual transcriptional activation, and 35S::*AcHSFA2–2* lines show significantly enhanced root vitality.
- Heat acclimation specifically induces *AcLBD1*, which modulates the mutual amplification cascade of *AcHSFA2–1* and *AcHSFA2–2* to enhance heat resilience.

## 1 Introduction

Frequent high-temperature events exert severe impacts on global plant growth and development. Investigations into the mechanisms underlying plant heat stress responses represent a classic yet hot area of research (reviewed in Kan et al., 2023; Staacke et al., 2025). The photosynthetic apparatus is highly sensitive to heat stress and constitutes a major focus in heat stress research. Elevated temperatures trigger reactive oxygen species (ROS) bursts, leading to oxidative stress, damage to cellular membranes, the electron transport chain, and photosynthetic structures, thereby inhibiting or arresting plant growth and development (Kan et al., 2023). During heat stress, increases in both air and soil temperature inevitably affect root development. The root system plays crucial roles in anchorage, water and nutrient uptake throughout the life cycle of terrestrial plants (Ye et al., 2025). How high temperatures modulate root vitality, particularly in succulent, shallow-rooted perennial species, is critical for plant thermotolerance. While the effects of heat on aboveground plant parts are well-documented, root responses to high temperatures remain understudied.

**Figure 1:**
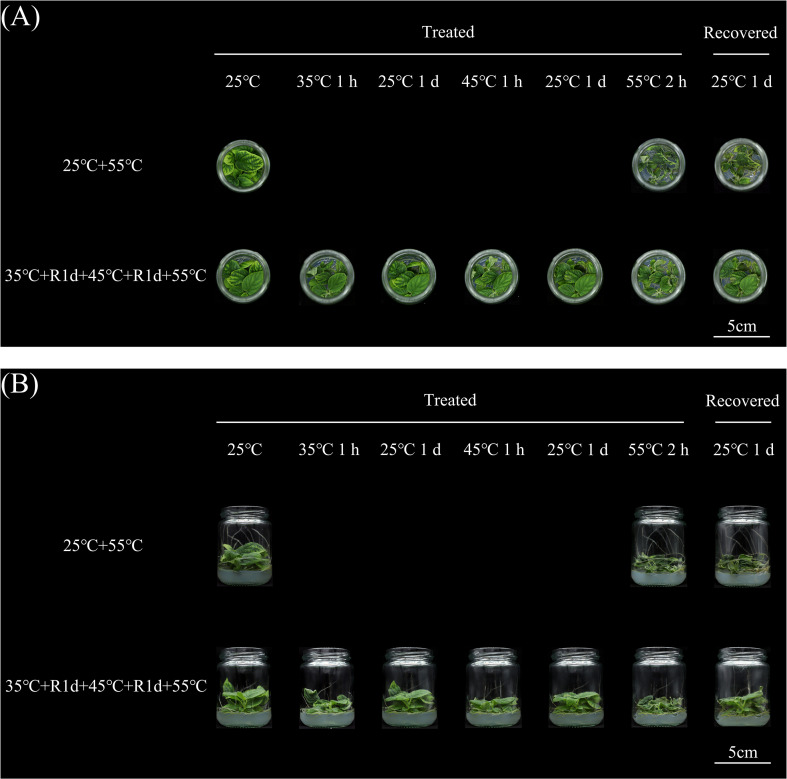
The effects of different heat treatments on kiwifruit plantlets growth were evaluated. The photographs of top-down **(A)** and horizontal view **(B)** had been taken during the treatments. The plantlets cultured in 25 °C were set as control. The heat stress (HS) carried out with 55 °C 2 h was recorded as 25°C+55°C. The heat acclimation (HA) recorded as 35 °C+R1d+45°C+R1d+55°C was carried out sequentially with 35 °C 1 hour (h), recovered in 25 °C 1 day (R1d), 45 °C 1 h, another R1d, 55 °C 2 h. All of these samples were cultured under 25 °C for 1 more day after different treatments.

Heat acclimation (HA), a pretreatment with mild heat stress that establishes thermomemory to enhance tolerance to subsequent severe heat, significantly improves thermotolerance in *Arabidopsis* seedlings (Ling et al., 2018). The heat shock transcription factor *AtHSFA2* is indispensable for the establishment of transcriptional heat stress memory (Lämke et al., 2016; Yamaguchi et al., 2021). Here, we demonstrate that HA-induced thermotolerance is conserved in kiwifruit, a typical succulent, shallow-rooted perennial fruit tree (**Figure 1**). Our previous work identified *AcHSFA2–1* as a regulator of extreme heat stress response (HSR) in kiwifruit (Shen et al., 2023). However, whether and how *AcHSFA2–1* responds to HA remains unknown.

The Lateral Organ Boundaries Domain (LBD) genes encode a plant-specific transcription factor family essential for lateral organ development in higher plants. For instance, *Arabidopsis* AtLBD11 acts as a key downstream transcription factor of cytokinins to promote root growth (Ye et al., 2025), and its degradation is critical for salt stress-mediated root growth inhibition (Dang et al., 2025). As anticipated, the functional diversity of the LBD family extends beyond lateral organ boundary formation (reviewed in Xu et al., 2016). For example, *MdLBD37* is involved in the heat-mediated repression of anthocyanin biosynthesis in apple fruits (Li et al., 2025). It suggested the potential of LBD in heat stress response. However, studies on LBD functions in temperature stress responses are still limited. Furthermore, the mechanism by which LBD transcription factors regulate root vitality under high temperatures remains unclear.

In this study, the heat-sensitive kiwifruit were used as the experimental material to evaluate the effects of HA on their physiological phenotypes and molecular responses. We found that HA-enhanced root vitality is a key factor driving the rapid recovery of net photosynthetic rate and the significant improvement of thermotolerance post-heat-stress. *AcHSFA2-2*, which clusters with *AcHSFA2–1* and both of them are homology of AtHSFA2 of *Arabidopsis*, participates in root vitality regulation. Moreover, *AcHSFA2–2* and *AcHSFA2–1* form a mutual amplification regulation loop. *AcLBD1*, which is strongly induced by HA treatment, cascades and amplifies the mutual amplification loop of AcHSFA2s by significantly enhancing *AcHSFA2–1* expression, thereby increasing the abundance of heat-protective HSP20 proteins and ultimately enhancing plant thermotolerance.

## 2 Materials and methods

### 2.1 Plant materials

To investigate the effects of heat stress on the growth and development of kiwifruit (*Actinidia chinensis* cv. Donghong), tissue-cultured plantlets were used as experimental materials. All of plantlets were first grown to the 5-leaf stage in a tissue-culture room maintained at 25 °C with a 16 h light/8 h dark photoperiod, and 5000 lux light intensity, to ensure uniform growth and health.

#### 2.1.1 *achsfa2–1* loss-of-function mutants plantlets

To generate the loss-of-function mutant *achsfa2-1*, potential off-target sites of target sequences were predicted using CRISPR-P v2.0 (http://cbi.hzau.edu.cn/crispr/), a plant-specific sgRNA design tool. Two sgRNAs (**Supplementary Table 1**) were designed to target specific sites at the start of the first exon in the coding region, aiming to induce premature termination of translation.

The designed sgRNAs were recombined with the pDE-KRS vector and introduced into *Agrobacterium tumefaciens* strain EHA105 via the freeze-thaw method. The methods for transformation and propagation were identical to Shen et al. (2023). Successfully transformed plants were selected by PCR. Genomic DNA was extracted, and a ~300-bp DNA fragment containing the sgRNA target regions was amplified with Hi-TOM primer pair (**Supplementary Table 1**). Mutations were further confirmed by sequencing using the Hi-TOM platform (China National Rice Research Institute, Chinese Academy of Agricultural Sciences, Hangzhou, China).

#### 2.1.2 35S::*AcHSFA2–2* kiwifruit lines plantlets

The open reading frame (ORF) of *AcHSFA2-2*, with its stop codon deleted, was cloned into the pSAK277 vector harboring a C-terminal FLAG tag, with the primers in **Supplementary Table 2**. The recombinant pSAK277-*AcHSFA2-2*-FLAG plasmid was introduced into *A. tumefaciens* strain EHA105. Once transgenic plantlets were established, leaf tissues were collected from WT and transgenic lines at the same developmental stage for genomic DNA (gDNA) and total RNA extraction. Positive transgenic plants were validated at both the DNA and RNA levels.

For DNA-level validation, the PCR amplification was carried out, with a forward primer been designed to target the CaMV 35S promoter, and a reverse primer specific to the ORF of *AcHSFA2-2* (**Supplementary Table 1**). PCR products were separated by agarose gel electrophoresis, visualized, and photographed to confirm the integration of the CaMV 35S promoter-*AcHSFA2–2* cassette into the kiwifruit genome.

The relative expression of *AcHSFA2–2* in the potential transgenic lines were detected by real-time quantitative PCR (RT-qPCR), with primers in **Supplementary Table 3**. Total RNA was extracted from whole plants using the CTAB method (Wang et al., 2022) and verified by agarose gel electrophoresis. First−strand cDNA was synthesized using the PrimeScript RT Reagent Kit with gDNA Eraser (Takara). qPCR primers were designed using Primer3Plus and validated by melting curves and sequencing. RT−qPCR was performed on a CFX96 system (Bio−Rad) using TB Green Premix Ex Taq II (Takara), with *Actin2* as the internal reference (Fullerton et al., 2020).

#### 2.1.3 35S::*AcHSFA2–2* plants

The 5-leaf stage of wild type ‘Donghong’ and 35S::*AcHSFA2–2* lines plantlets were transplanted into pots (11 cm inner diameter) containing a peat soil: vermiculite: perlite mixture (5:1:1, v/v/v). Plants were grown in a growth chamber at 25 °C, 60-70% relative humidity, 5000 lux light intensity, and a 16 h/8 h light/dark photoperiod for 3 months.

### 2.2 Heat acclimation treatment

The heat stress treatment has been carried out with the 5-leaf stage of wild type ‘DH’. Glass culture vessels containing these plantlets were first uncapped and incubated at 25 °C for 1 h, then subjected to treatment. For heat acclimation treatments, constant−temperature illuminated incubators were used for 35 °C, 45 °C, and 55 °C exposures, while the tissue−culture incubator provided the 25 °C control environment.

In the 25 °C + 55 °C group, plantlets were directly exposed to 55 °C for 2 h and then returned to 25 °C for 1 day of recovery. In the 35 °C + R1d + 45 °C + R1d + 55 °C group, plantlets were sequentially treated at 35 °C for 1 h (recovery at 25 °C for 1 day), 45 °C for 1 h (recovery at 25 °C for 1 day), and 55 °C for 2 h (recovery at 25 °C for 1 day).

### 2.3 Heat stress treatment

The heat stress treatment has been carried out with the 5-leaf stage of wild type ‘DH’, achsfa2-1, as well as 35S::*AcHSFA2–2* lines. Glass culture vessels containing these plantlets were first uncapped and incubated at 25 °C for 1 h, then subjected to heat treatment at 45 °C for 4 h, after which they were recapped and returned to 25 °C for a 9-day recovery period. Plantlets that were maintained in the 25 °C with uncapped cultured for 4 h served as the control group.

All the heat acclimation and heat stress treatments included at least three independent biological replicates, with ≥3 plants per replicate. Plant phenotypes were photographed during treatment and recovery.

### 2.4 Measurement of photosynthetic parameters

Photosynthetic parameters were measured on the 2nd or 3rd fully expanded leaves from the base. Remaining tissues were immediately frozen in liquid nitrogen and stored at −80 °C for further use.

Net photosynthetic rate (Pn), stomatal conductance (Gs), transpiration rate (Tr), and intercellular CO_2_ concentration (Ci) were determined using a LI−6800 portable photosynthesis system (LI−COR, Beijing, China). Light intensity was matched to that of the tissue−culture incubator. Leaf chamber conditions were set as follows: CO_2_ concentration 400 μmol·mol^-^¹, relative humidity 50%, airflow rate 500 μmol·s^-^¹, and temperature automatically adjusted to ambient. Measurements were recorded after 3–5 min of stabilization.

### 2.5 Root vitality assay

Root vitality was measured using a modified 2, 3, 5−triphenyltetrazolium chloride (TTC) method (Chen et al., 2024). The 0.25 g fresh root tissue was incubated in 5 mL 0.4% TTC solution mixed with an equal volume of 0.1 M phosphate buffer. For blank controls, 2 mL 1 M H_2_SO_4_ was added before tissue immersion. Samples were incubated at 37 °C in darkness for 2 h, after which the reaction was terminated with 2 mL 1 M H_2_SO_4_. Roots were blotted dry, ground in ethyl acetate to extract formazan, and the volume adjusted to 10 mL. Absorbance was measured at 485 nm against the blank, and TTC reduction was quantified using a standard curve. Root vitality was calculated as:


Root vitality=TTC reduction (mg)/[root fresh weight (g)×time (h)]

### 2.6 Subcellular localization

The full−length ORFs of *AcLBD1* and *AcHSFA2–2* were cloned into the eGFP vector, with the primers listed in **Supplementary Table 2**, and transformed into *A. tumefaciens* strain GV3101, respectively. Recombinant strains were mixed 1:1 (v/v) with an mCherry−containing strain and infiltrated into *Nicotiana benthamiana* leaves. Empty eGFP vector mixed 1:1 with mCherry served as a control. Infiltration buffer contained 150 μM acetosyringone, 10 mM MES, and 10 mM MgCl_2_ (OD_600_ = 0.75). After 3 days, GFP and mCherry fluorescence in the mesophyll cells was observed using a Zeiss LSM 900 confocal laser scanning microscope (Zeiss, Jena, Germany).

### 2.7 Dual−luciferase assays

The full−length ORFs of *AcLBD1* and *AcHSFA2–2* were cloned into pGreen II 0029 62−SK, with the primers listed in **Supplementary Table 2**, and transformed into GV3101, respectively. Promoters of *AcHSFA2–1* and *AcHSFA2–2* were cloned into pGreen II 0800−LUC, with the primers listed in **Supplementary Table 2**. The *AcHSFA2–1* effector (pGreen II 0029 62−SK) and *AcHSP20-1/-2/-3* promoter reporters (pGreen II 0800−LUC) were from a previous study (Shen et al., 2023).

*Agrobacteria* were resuspended in the same buffer as for subcellular localization. Effector and reporter strains were mixed 10:1 (v/v) and infiltrated into *N. benthamiana* leaves. Empty vectors mixed with reporters (10:1) served as controls. After 3 days, firefly luciferase (LUC) and Renilla luciferase (REN) activities were measured using a Dual Luciferase Reporter Assay Kit (YEASEN, Shanghai, China). Trans-activation vitality was expressed as the LUC/REN ratio. At least three independent experiments with three biological replicates were performed.

### 2.8 Electrophoretic mobility shift assays

The *AcHSFA2–2* ORF was cloned into pGEX4T−1, with the primers listed in **Supplementary Table 2**, and expressed in *Escherichia coli* BL21 (DE3). Cells were grown in LB medium (100 μg·mL^-^¹ ampicillin, 34 μg·mL^-^¹ chloramphenicol) to OD_600_ = 0.6, then induced with 0.5 mM IPTG at 16 °C for 20 h. Recombinant AcHSFA2−2−GST protein was purified using a GST−tag Protein Purification Kit (Beyotime, China). 5′−biotin−labeled and unlabeled (cold) probes for AcHSP20-1/-2/-3 promoters were from a previous study (Shen et al., 2023). Probes were annealed to double strands. EMSA was performed using a Chemiluminescent EMSA Kit (Beyotime, China).

### 2.9 Phylogenetic analysis and sequence alignment

Amino acid and promoter sequences were retrieved from the kiwifruit genome database (https://kiwifruitgenome.org/). *Arabidopsis* LBD sequences were obtained from published data (https://academic.oup.com/plphys/article/129/2/747/6110259). Multiple sequence alignment was performed using DNAMAN, and phylogenetic trees were constructed with MEGA11. *Cis*−element analysis LBD binding motifs and heat−responsive *cis*−elements were predicted using PlantCARE and published references (https://www.tandfonline.com/doi/full/10.1128/MCB.18.11.6340; https://academic.oup.com/nar/article/35/19/6663/2402815; https://academic.oup.com/plcell/article/23/10/3671/6098232). Results were visualized using TBtools.

### 2.10 Statistical analysis

Graphs were generated using the R ggplot2 package, GraphPad Prism v.10.1.2, or Adobe Photoshop 2024. Student’s *t*−test and Tukey’s multiple comparison test were performed using SPSS (P < 0.05).

## 3 Results

### 3.1 Heat acclimation enhances tolerance to extreme heat stress in kiwifruit plants

To explore the mechanisms underlying plant responses to heat stress, this study designed a heat acclimation (HA) regime that mimics natural temperature fluctuations, involving a gradual transition from mild to extreme heat stress.

Visual assessment of leaf curling and stem erectness showed that, relative to control plants, heat−acclimated plants exhibited milder leaf margin curling and retained a more expanded leaf phenotype upon exposure to 55 °C for 2 h (**Figure 1A**). However, HA had no significant effect on stem erectness, as both control and acclimated plants displayed a pronounced reduction in stem rigidity under 55 °C stress (**Figure 1B**). Following the cessation of extreme heat stress, the recovery capacity of the two groups differed markedly at 25 °C. Control plants showed no obvious alleviation of leaf curling or stem wilting. By contrast, acclimated plants exhibited fully expanded leaves, rapid restoration of stem erectness, and a swift recovery of growth vigor (**Figure 1**). These results confirm that HA significantly enhances plant thermotolerance.

### 3.2 Heat acclimation promotes recovery of leaf net photosynthetic rate and root vitality

**Figure 2:**
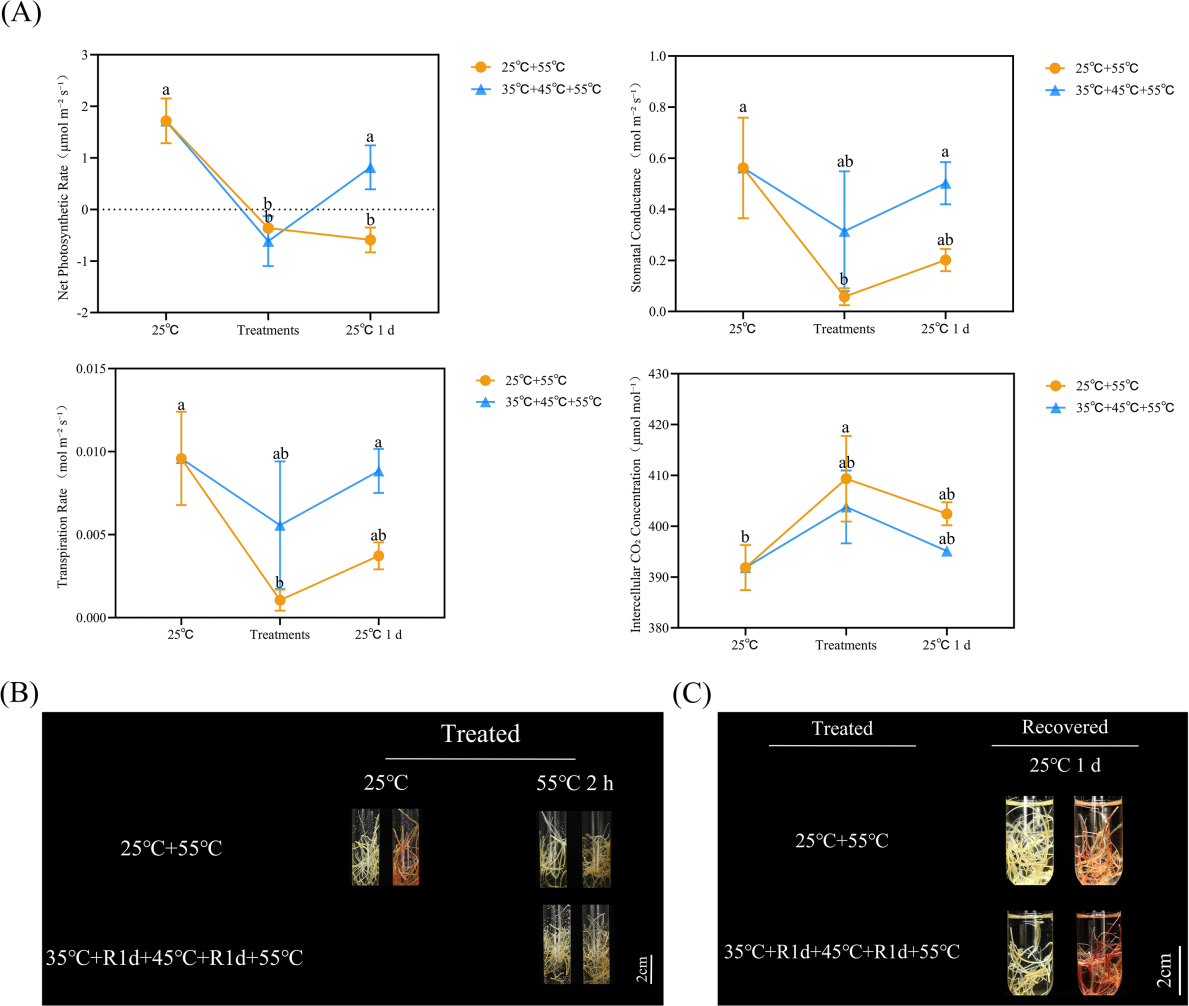
Comparison of physiological index changes in plantlets under different treatments. **(A)** Determination of net photosynthetic rate, stomatal conductance, transpiration rate and intercellular CO_2_ concentration in the leaves. Where the 25 °C mean the indices of leaves grown under 25 °C conditions; the Treatments indicated the indices after HS (25°C+55°C, orange lines) or HA (35°C+R1d+45°C+R1d+55°C, blue lines) treatments; the 25 °C 1d represented the indices of leaves recovered for 1 day at 25 °C after the above treatments. **(B, C)** Evaluation of root activities after HS and HA treatments **(B)**, and after 1 day of recovery growth at 25 °C **(C)**. Roots in the left tubes in each biological replicate were the blank controls, and the right tubes were the tests with 1 h incubated reaction. Each treatment was carried out with three biological replicates, and each biological replicate included at least two kiwifruit plantlets. Error bars represent ± SE from three biological replicates. Different letters indicate the significant differences which were statistically analyzed by Duncan method.

The photosynthetic parameters, including net photosynthetic rate (Pn), stomatal conductance (Gs), transpiration rate (Tr), and intercellular CO_2_ concentration (Ci), as well as root vitality, were further quantified. HA significantly improved the post−heat-stress recovery of leaf Pn. Under 55 °C for 2 h, control plants exhibited a sharp decline in Pn, Gs, and Tr, accompanied by a significant increase in Ci (**Figure 2A**). Although acclimated plants also showed reduced Pn, their Gs, Tr, and Ci remained unchanged (**Figure 2A**). After stress relief, Gs, Tr, and Ci in control leaves returned to levels comparable to those at 25 °C, but Pn remained significantly depressed (**Figure 2A**). In contrast, Pn in acclimated leaves fully recovered to pre−stress levels (**Figure 2A**). These findings indicate that changes in Gs, Tr, and Ci are not the primary cause of the rapid Pn recovery induced by HA.

Root vitality was then evaluated using 2, 3, 5−triphenyltetrazolium chloride (TTC) staining, where deeper red coloration indicates higher vigor. Similar to Pn, root vitality in both groups decreased significantly under 55 °C stress (**Figure 2B**). Within 1 day of recovery, root vitality in acclimated plants was fully restored to the 25 °C control level, whereas that in control plants remained significantly lower (**Figure 2C**).

These results demonstrate that the recovery of root vitality is a key determinant underlying the rapid restoration of leaf net photosynthetic rate following heat stress.

### 3.3 Heat acclimation significantly enhances the transcript abundance of *AcLBD1*

**Figure 3:**
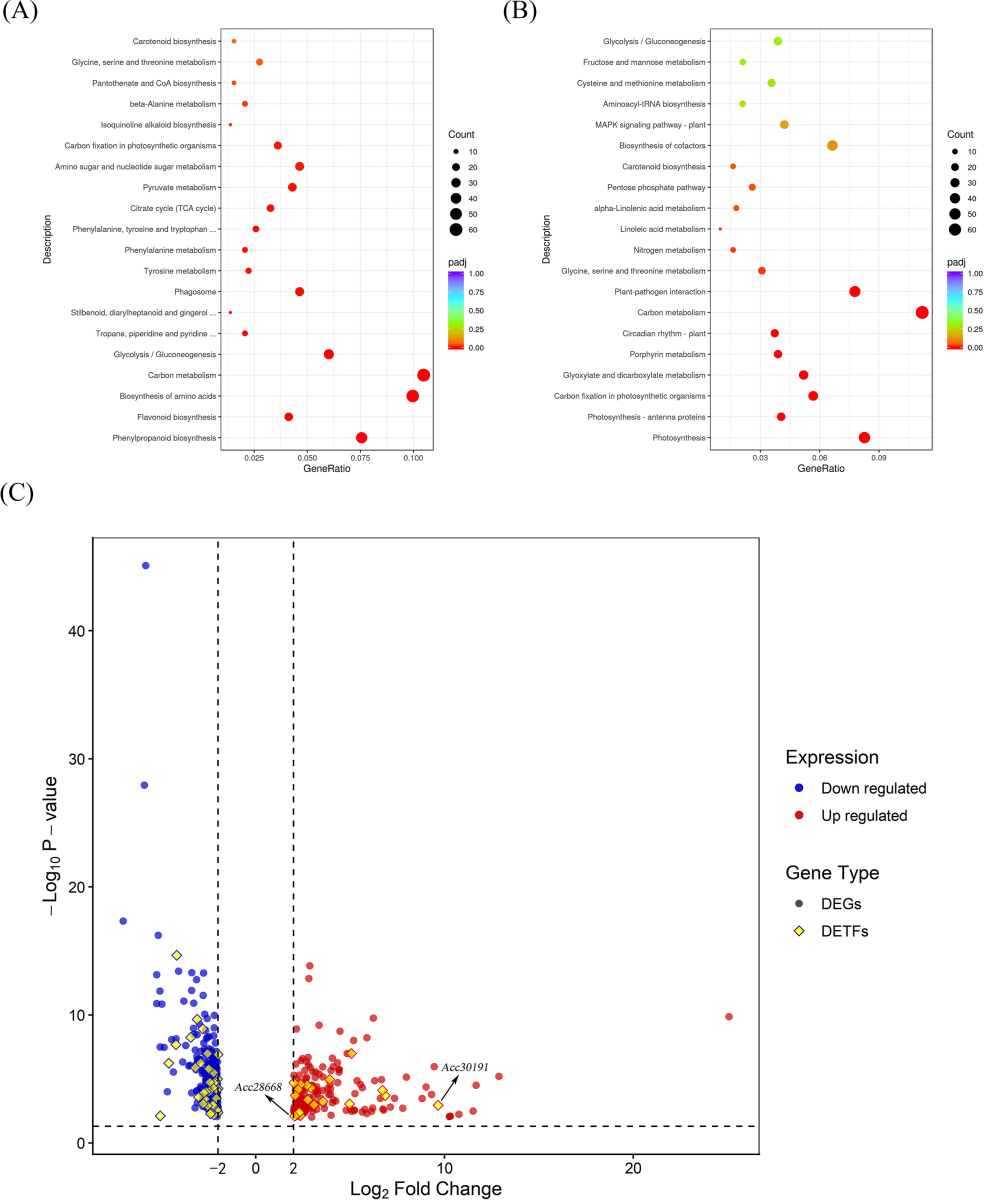
Transcriptome analysis of kiwifruit leaves under heat acclimation treatment. **(A, B)** GO enrichment analysis of the up-regulated **(A)** and down-regulated **(B)** differentially expressed genes (DEGs) in heat-acclimated leaves. **(C)** Volcano plot showing the fold change of DEGs. Blue and red dots represent down-regulated and up-regulated DEGs, respectively, and diamonds represent differentially expressed transcription factors (DETFs).

Transcriptomic libraries were generated from leaves of control and heat−acclimated plants following exposure to 55 °C for 2 h. Differentially expressed genes (DEGs) were identified using the thresholds of |log_2_FC| ≥ 1.8, MaxFPKM ≥ 5 and *pvalue<* 0.05. Gene Ontology (GO) enrichment analysis revealed that upregulated DEGs in the acclimated group were significantly enriched in pathways including phenylpropanoid biosynthesis, amino acid biosynthesis, and carbon metabolism (**Figure 3A**), whereas downregulated DEGs were predominantly associated with photosynthesis, plant−pathogen interaction, and carbon metabolism (**Figure 3B**).

Analysis of expression fold−changes identified *AcLBD1* (*Acc30191*) as the transcription factor most strongly induced by HA (**Figure 3C**). Additionally, *AcHSFA2-1* (*Acc28668*), a key heat−shock transcription factor regulating extreme heat stress response in kiwifruit identified in our previous work (Shen et al., 2023), also responded significantly to HA. Both genes exhibited markedly elevated transcript levels following acclimation, with *AcLBD1* showing the highest induction (**Figure 3C**). These findings suggest that *AcLBD1* plays an indispensable role in the heat−acclimation response of kiwifruit.

### 3.4 AcLBD1 exhibits the potential to respond to temperature fluctuations and transcriptionally activate target genes

**Figure 4:**
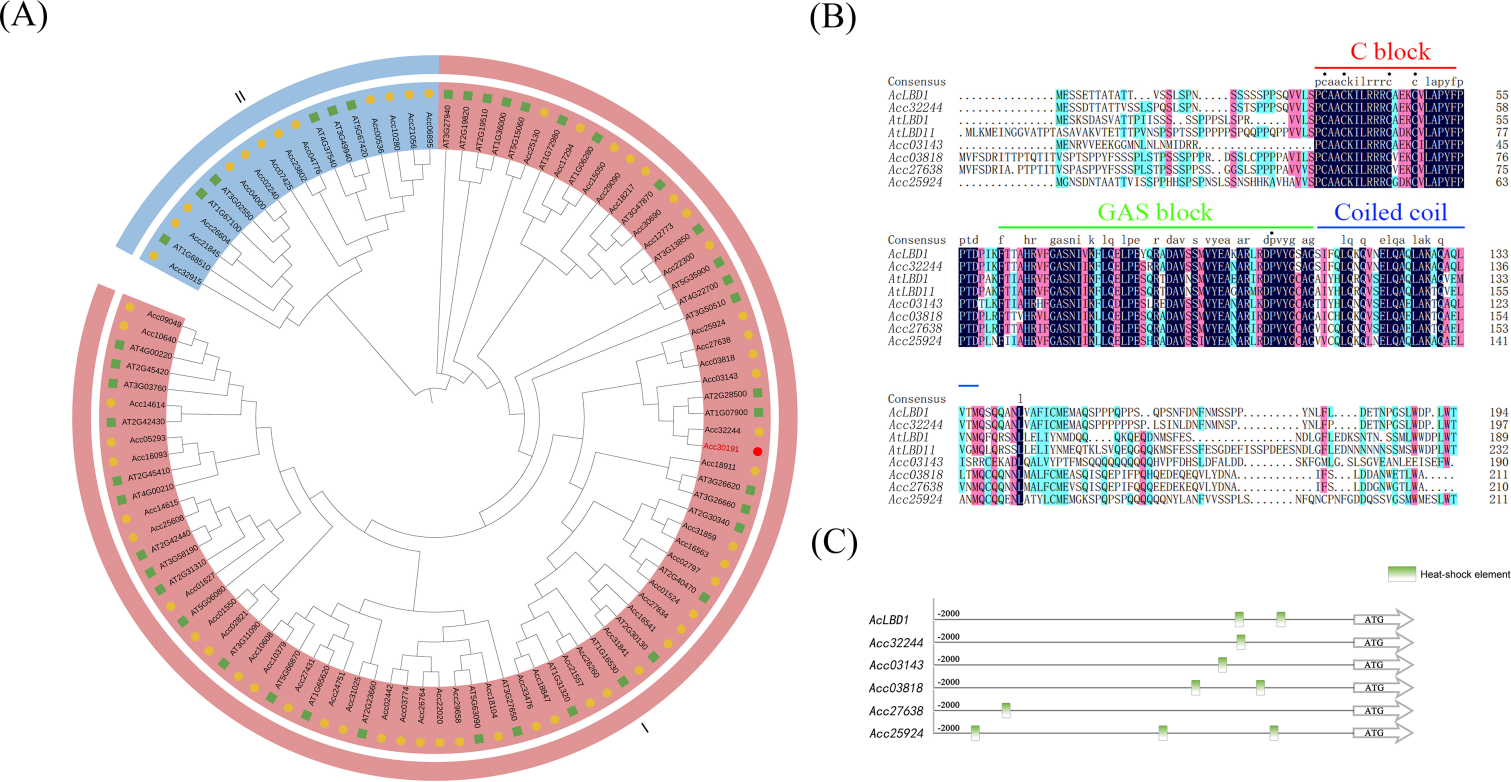
Bioinformation analysis of LBD family members. **(A)** Phylogenetic tree analysis showing the classification of LBDs into two major subfamilies, with the Subfamily I highlighted in red and Subfamily II in blue. Green squares represent LBD members in *Arabidopsis thaliana*, and yellow circles represent LBD members in *Actinidia chinensis*. **(B)** Sequence alignment of LBD members in the same clade as AcLBD1. Three conserved blocks in the LBD domain were marked: C block, GAS block, and Coiled coil. **(C)** Analysis of *cis*-acting elements in the promoters of six LBD members in the same clade as AcLBD1.

To dissect the molecular mechanism underlying *AcLBD1* function in HA, we performed phylogenetic and sequence feature analyses to predict its functional mode. Phylogenetic tree analysis showed that Acc30191 clustered in subgroup I of the LBD family, with the highest homology to *Arabidopsis* AtLBD1 (AT1G07900) and AtLBD11 (AT2G28500) (**Figure 4A**). Accordingly, *Acc30191* was designated *AcLBD1*.

Among the 59 LBD members in kiwifruit, six clustered in the same clade as AtLBD1 and AtLBD11 (**Figure 4A**). Amino acid sequence alignment revealed high specificity in the N− and C−terminal regions of LBD proteins, while the middle region was relatively conserved. Similar to AtLBD1/11, AcLBD1 and other kiwifruit LBD members in this clade contained three conserved domains in the middle region: the C Block (CX_2_CX_6_CX_3_C), GAS Block, and Coiled−coil (LX_6_LX_3_LX_6_L) (**Figure 4B**). The C Block is essential for DNA binding and transcriptional activation by LBD proteins (Kong et al., 2017).

Further analysis of *cis*−acting elements in the promoters of these six *AcLBDs* revealed that each contained at least one heat−shock element (HSE), and the *AcLBD1* promoter harbored two HSEs (**Figure 4C**). HSEs are key regulatory elements in the heat−shock response and represent core targets for dissecting thermotolerance mechanisms (Lou et al., 2025). The presence of HSEs provides a sequence basis for the responsiveness of *AcLBD1* to heat acclimation.

### 3.5 AcLBD1 transcriptionally activates the AcHSFA2-1−AcHSP20 module

**Figure 5:**
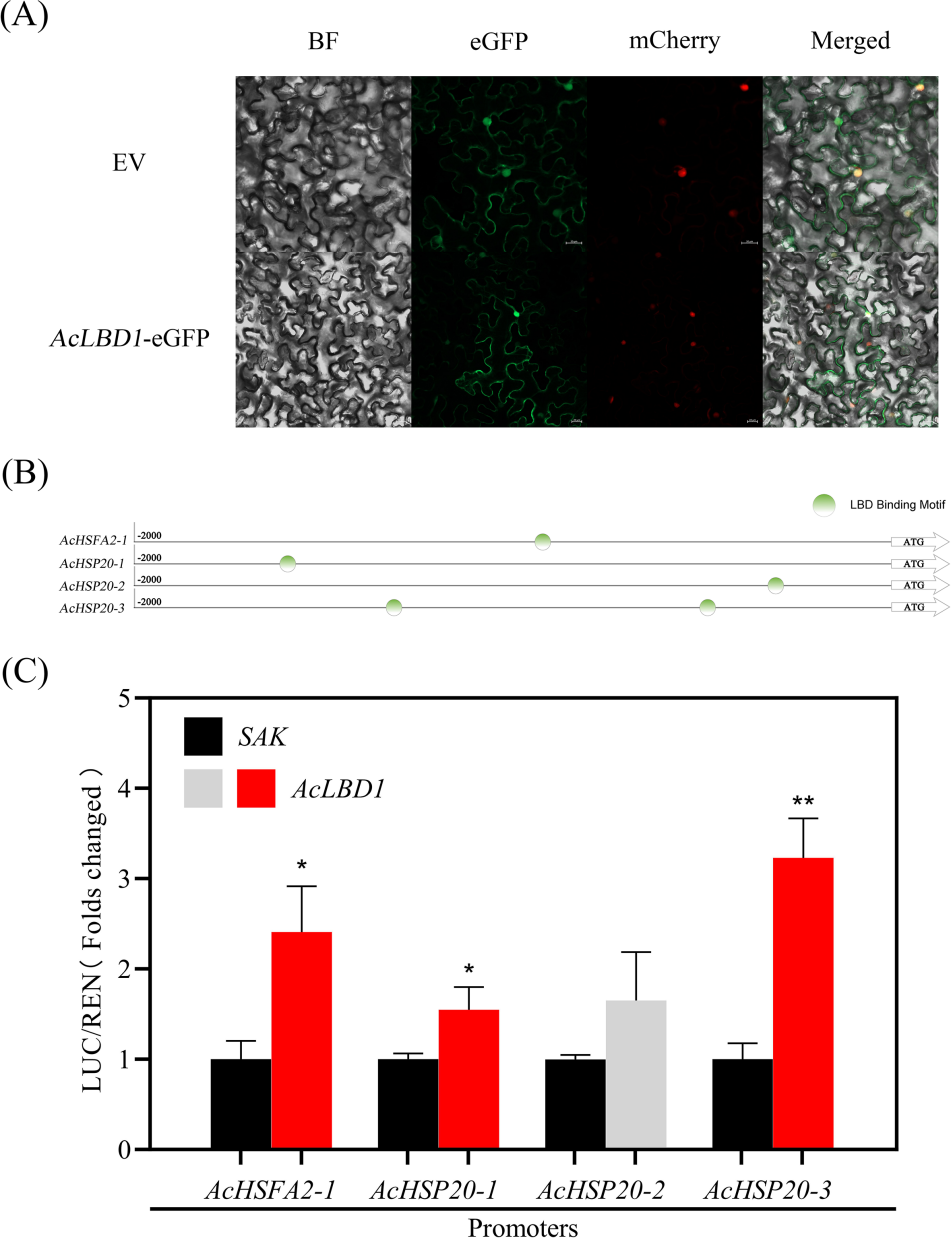
Analysis of the transcriptional regulatory characteristics of AcLBD1. **(A)** Subcellular localization analysis of AcLBD1. EV represents the empty vector control, BF Bright-field, eGFP enhanced green fluorescent protein, mCherry the red fluorescent protein localized exclusively in the nucleus, and Merged the overlay of the three images. **(B)** Prediction of cis-acting elements in the promoter sequences of *AcHSFA2-1*, *AcHSP20-1*, *AcHSP20–2* and *AcHSP20-3*. Circles indicate the LBD Binding Motif. **(C)** Analysis of the transcript regulation of AcLBD1 on the three genes promoter activities were carried out with dual luciferase assays. The ratio of LUC to RNE of the empty vector was set as 1 for *AcHSFA2-1*, *AcHSP20-1*, *AcHSP20–2* and *AcHSP20–3* promoters, respectively. At least three independent experiments had been taken to confirm the significant regulatory effect. Error bars represent ± SE from three biological replicates in each experiment. The statistical analysis were carried out with Students’ *t*-test. Asterisks indicate significant differences of relative value of LUC/REN of the TFs compared to the SAK. (*P< 0.05; **P< 0.01).

Subcellular localization analysis showed that AcLBD1 was localized to both the plasma membrane and nucleus (**Figure 5A**), indicating that AcLBD1 functions as a typical transcription factor in the nucleus.

Based on their co−expression patterns during HA, we hypothesized that *AcLBD1* acts upstream of *AcHSFA2-1*. *Cis*−element analysis confirmed the presence of a canonical LBD binding motif [(G)CGGC(G)] in the *AcHSFA2–1* promoter (**Figure 5B**). Similarly, the promoters of *AcHSP20-1*, *AcHSP20-2*, and *AcHSP20-3*—all known targets of AcHSFA2−1—also contained LBD binding motifs (**Figure 5B**). Dual−luciferase assays verified that AcLBD1 significantly activated the promoters of *AcHSFA2-1*, *AcHSP20-1*, and *AcHSP20-3*, with fold−induced of 2.41, 1.55, and 3.23, respectively (**Figure 5C**).

### 3.6 *achsfa2–1* mutants exhibit complete loss of thermotolerance

Given that AcLBD1 mediates HA via transcriptional activation of the AcHSFA2-1-AcHSP20 module, the *achsfa2–1* loss−of−function mutants was generated using CRISPR−Cas9 to further dissect the functional mechanism of *AcLBD1*.

**Figure 6:**
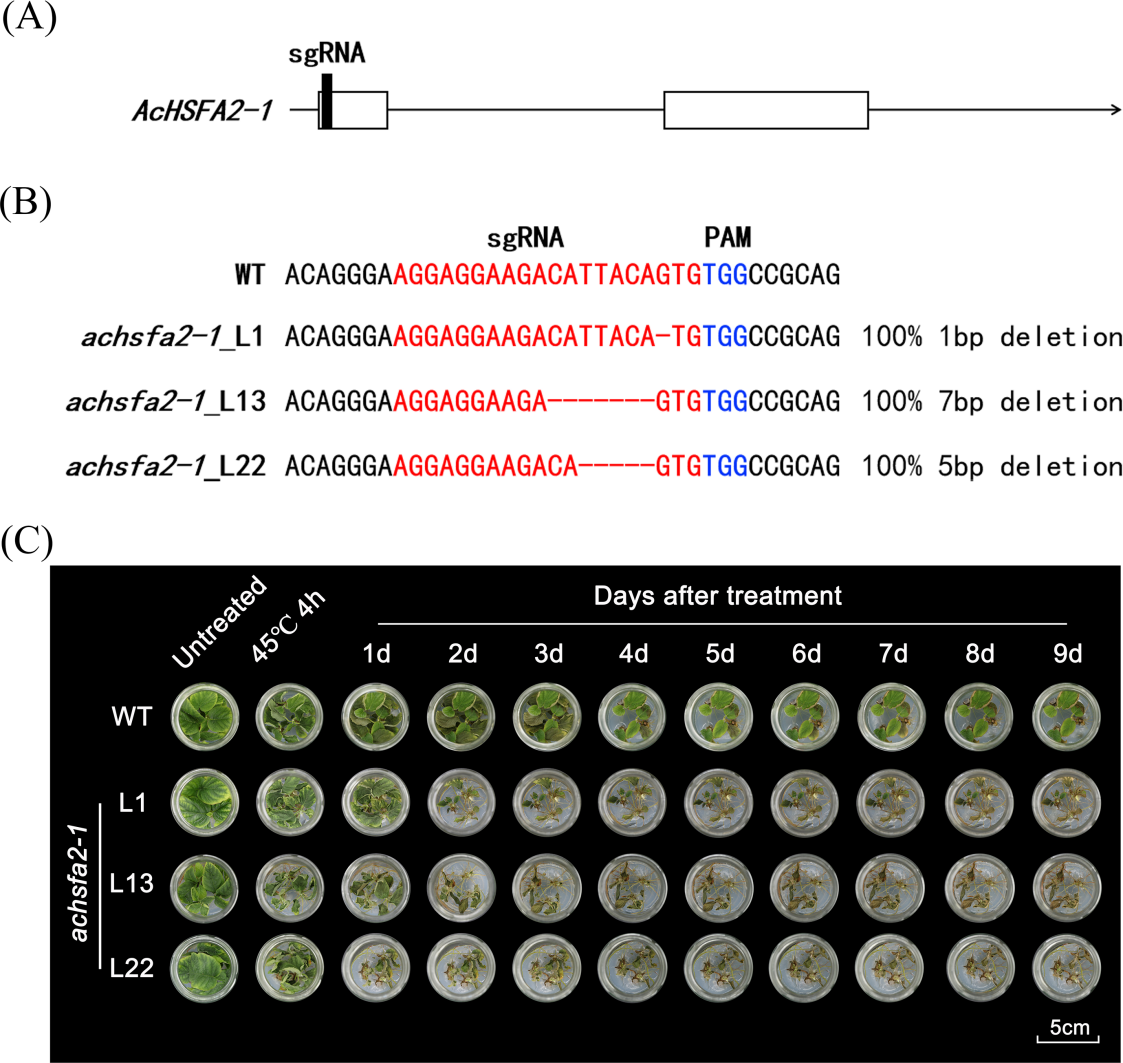
Generation and thermotolerance analysis of *achsfa2–1* loss-of-function mutants. **(A)** Schematic diagram showing the localization of sgRNA in the genomic sequence of *AcHSFA2-1*, where rectangles represent exons and arrows represent introns, respectively. **(B)** Sequence mutation analysis of three *achsfa2–1* mutants by HiTom sanger sequencing. WT represents wild-type plantlets, and the three *achsfa2–1* mutants are designated as L1, L13 and L22; the red nucleotide sequences indicate the sgRNA regions. **(C)** Evaluation of thermotolerance in WT and three *achsfa2–1* mutants under heat stress treatment (45°C for 4 h). After heat stress, all plantlets were transferred to 25°C for recovery growth, and phenotypic images were captured at the designated time points during the treatment.

The genomic sequence of *AcHSFA2–1* contains two exons, and the sgRNA was designed to target the first exon near the start codon (**Figure 6A**). Hi−Tom sequencing confirmed effective base−deletion mutations in the target region of three independent *achsfa2–1* lines: Line1, Line13, and Line22 harbored 1−bp, 7−bp, and 5−bp deletions, respectively, resulting in premature termination of the coding sequence and loss of gene function (**Figure 6B** and **Supplementary File 1**).

Thermotolerance was assessed following exposure to 45 °C for 4 h. All three *achsfa2–1* lines displayed a complete loss of thermotolerance. Compared with wild−type (WT) plants, the *achsfa2–1* exhibited severe leaf curling and wilting during heat stress, and gradually withered and died even after stress relief (**Figure 6C**). While the WT plants partially recovered during post-heat-stress. These results demonstrate that *AcHSFA2–1* is essential for thermotolerance in kiwifruit.

Transcriptomic profiling of WT and *achsfa2–1* leaves under 45 °C for 4 h revealed that the transcript abundance of thousands of genes was altered by *AcHSFA2-1*. Principal component analysis (PCA) and Pearson correlation analysis confirmed high data reproducibility among the three biological replicates for WT, *achsfa2–1* Line1, and Line13 (**Supplementary Figures 1A, B**). Compared with heat−stressed WT, Line1 and Line13 exhibited 4, 657 and 8, 940 DEGs, respectively, with 2, 623 up− and 2, 034 downregulated in Line1, and 4, 114 up− and 4, 826 downregulated in Line13 (**Supplementary Figure 1C**). A total of 3, 391 DEGs were shared between Line1 and Line13 under heat stress, of which 735 were also differentially expressed in Line22 (**Supplementary Figure 1D**).

### 3.7 AcHSFA2–1 and AcHSFA2–2 form a positive regulation feedback loop via mutual transactivation

Although thousands of genes were differentially expressed in heat−stressed *achsfa2–1* mutants, *AcLBD1* expression was unaffected, further confirming that AcLBD1 acts upstream of AcHSFA2-1. To refine the molecular regulatory network underlying *AcLBD1*-mediated HA, the DEGs from 35S::*AcHSFA2-1* (NCBI: PRJNA797274) and *achsfa2–1* transcriptomes were integrated to identify the key regulators.

**Figure 7:**
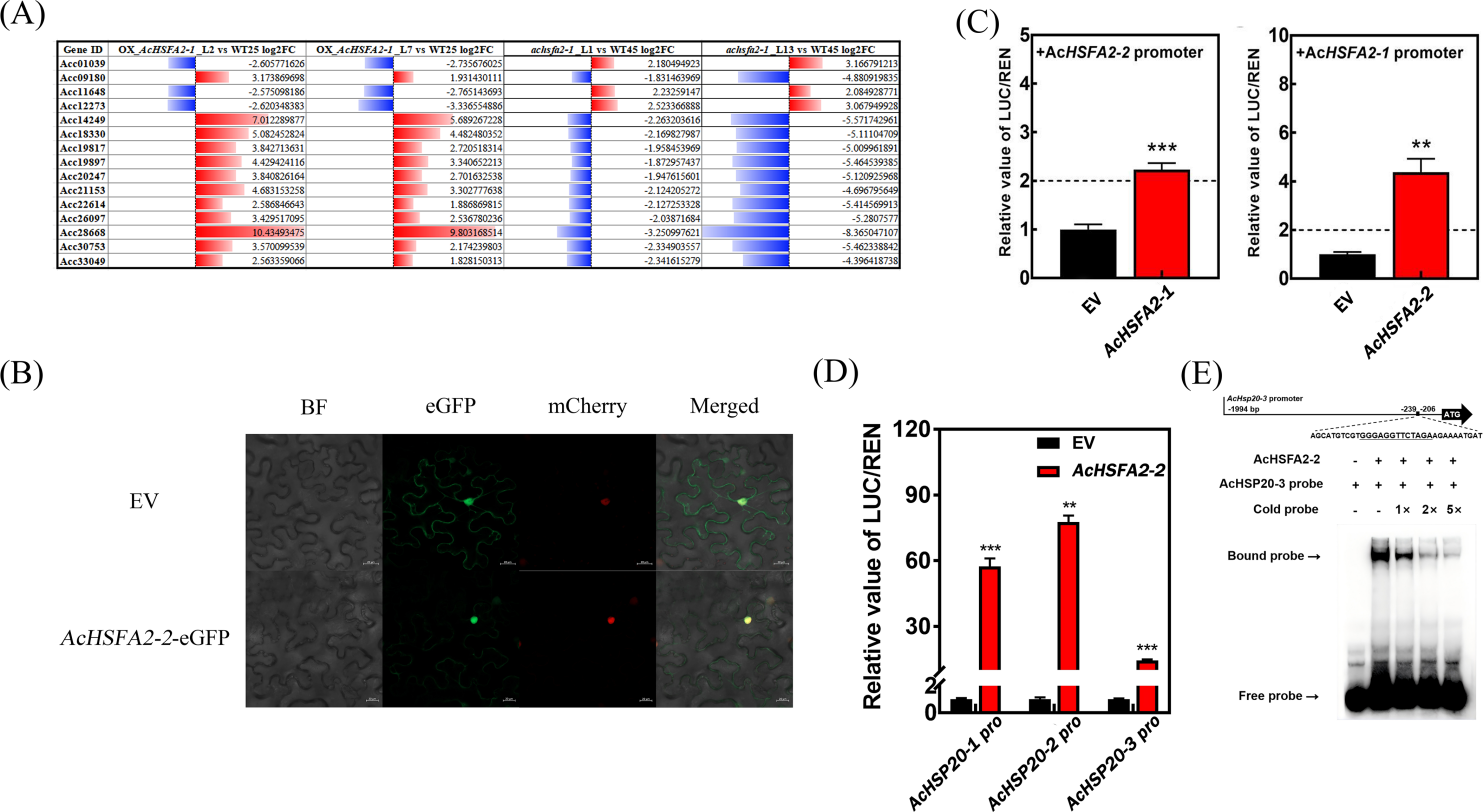
AcHSFA2–1 and AcHSFA2–2 form a positive regulatory loop. **(A)** Screening of differentially expressed genes (DEGs) with opposite expression trends in the transcriptomes of the 35S::*AcHSFA2–1* and the *achsfa2–1* mutant. **(B)** Subcellular localization analysis of AcHSFA2-2. EV represents the empty vector control, BF bright-field, eGFP enhanced green fluorescent protein, mCherry the red fluorescent protein localized exclusively in the nucleus, and Merged the overlay of the three images. **(C)** Verification of the mutual *trans*-activation regulatory effect between AcHSFA2–1 and AcHSFA2–2 via dual-luciferase reporter assay. **(D)** Analysis of the transcript regulatory effect of AcHSFA2–2 on the promoter activities of *AcHSP20-1*, *AcHSP20–2* and *AcHSP20-3*. The ratio of LUC to RNE of the empty vector was set as 1, respectively. At least three independent experiments had been taken to confirm the significant regulatory effect. Error bars represent ± SE from three biological replicates in each experiment. The statistical analysis were carried out with Students’ *t*-test. Asterisks indicate significant differences of relative value of LUC/REN of the TFs compared to the SAK. (*P< 0.05; **P< 0.01; ***p<0.001). **(E)** Electrophoretic mobility shift assay (EMSA) analysis of the binding effect of AcHSFA2–2 to the promoter sequence of *AcHSP20-3*. The cold probes were set with three concentration gradients. “-” represent the absence, “+” represent the presence.

DEGs were filtered using the criteria |log_2_FC| ≥ 1.8, MaxFPKM ≥ 5 and *pvalue*< 0.05, with an inverse expression pattern between 35S::*AcHSFA2-1* (Line2, Line7) and *achsfa2-1* (Line1, Line13). In total, 15 genes showed opposite expression trends across all four lines (**Figure 7A**), including two transcription factors: *AcHSFA2–1* and another HSF, *Acc22614* (**Figure 7A**). Phylogenetic analysis of AcHSF family members (Shen et al., 2023) revealed that Acc22614 is most closely related to AcHSFA2–1 and clusters in the same subgroup; thus, it was designated *AcHSFA2-2*.

Similar to AcHSFA2−1, AcHSFA2−2 localized to the nucleus, consistent with its function as a transcription factor (**Figure 7B**). Dual−luciferase assays demonstrated that AcHSFA2−1 significantly activated the *AcHSFA2–2* promoter vitality by 2.22−fold, while AcHSFA2−2 more strongly enhanced the *AcHSFA2–1* promoter by 4.37−fold (**Figure 7C**). Furthermore, AcHSFA2−2 *trans*-activated the promoters of three *AcHSP20s* (*AcHSP20-1*, *AcHSP20-2*, *AcHSP20-3*) by 57.03−, 77.32−, and 14.56−fold, respectively (**Figure 7D**), with activation magnitudes far exceeding those of AcLBD1 (**Figure 5C**).

To verify direct binding to *AcHSP20s* promoters, the 65.5−kDa AcHSFA2−2−GST recombinant protein was purified (**Supplementary Figure 2A**). Probes for the three *AcHSP20s* promoters were designed as previously described (Shen et al., 2023). EMSA showed that AcHSFA2−2 directly bound to HSE *cis*−elements in the *AcHSP20–2* and *AcHSP20–3* promoters (**Supplementary Figure 2B**). Competitive EMSA using unlabeled cold probes further confirmed specific binding of AcHSFA2−2 to Probe4 (GGAGGTTCT, -239 to -206 bp) in the *AcHSP20–3* promoter, with band intensity diminishing as cold probe concentration increased (**Figure 7E**). These results solidly validate the direct binding of AcHSFA2−2 to *AcHSP20* promoters.

Collectively, these findings indicate that AcHSFA2−2, like AcHSFA2−1, regulates kiwifruit thermotolerance by transcriptionally activating *AcHSP20s*. Moreover, AcHSFA2−1 and AcHSFA2−2 mutually *trans*-activate each other, forming a positive regulation feedback loop.

### 3.8 35S::*AcHSFA2–2* kiwifruit plants exhibit significantly enhanced root vitality

**Figure 8:**
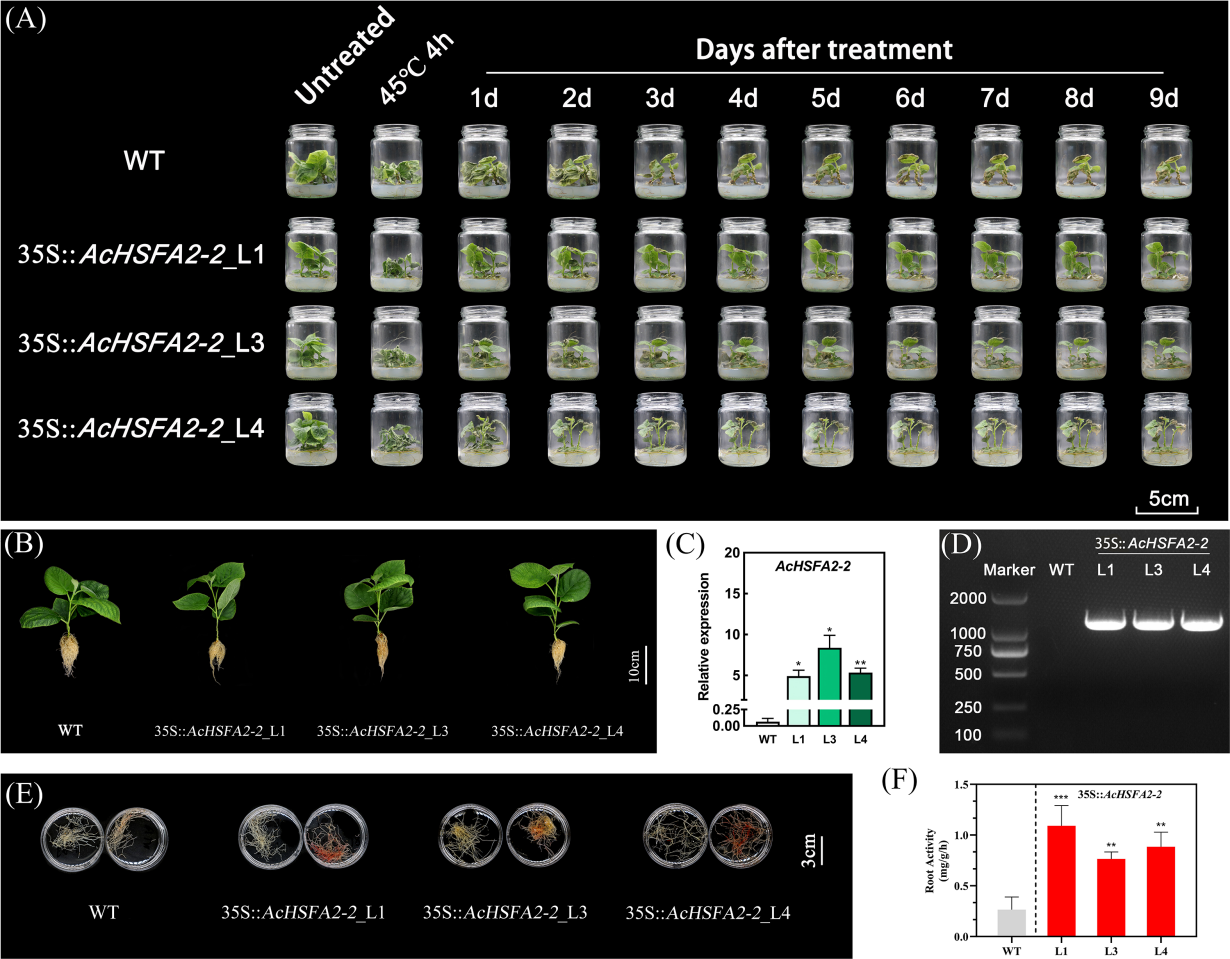
Over-expression of *AcHSFA2–2* significantly enhances heat tolerance and root vitality. **(A)** Phenotype changes of wild type and three lines of 35S::*AcHSFA2–2* kiwifruit under 45°C for 4 h and 25°C for 9 days. **(B)** Growth performance of wild-type and three 35S::*AcHSFA2–2* lines. The expression level and chromosomal integration of *AcHSFA2–2* in over-expression lines were verified by quantitative real-time PCR (qPCR) **(C)** and conventional PCR electrophoresis **(D)**. Qualitative **(E)** and quantitative **(F)** analysis of root vitality in wild-type and three 35S::*AcHSFA2–2* lines. Roots in the left in each biological replicate were the blank controls, and the right were the tests with 1 h incubated reaction. Error bars represent ± SE from three biological replicates. (*P< 0.05; **P< 0.01).

To characterize the biological function of *AcHSFA2–2* in planta, we generated three independent 35S::*AcHSFA2–2* kiwifruit lines (Line1, Line3, and Line4). Under 25 °C conditions, 35S::*AcHSFA2–2* plants displayed normal growth and development comparable to WT (**Figures 8A, B**), with no significant alterations in leaf chlorophyll content (data not shown).

Following exposure to 45 °C for 4 h, 35S::*AcHSFA2–2* plants fully recovered turgor and leaf expansion within 1 day, whereas WT plants partially recovered during the 9 days (**Figure 8A**). Relative to WT, *AcHSFA2–2* transcript levels were elevated by 75−, 129−, and 82−fold in the three over-expression lines, respectively (**Figure 8C**). The *AcHSFA2–2* open reading frame (ORF) driven by the CaMV 35S promoter was successfully integrated into the kiwifruit genome (**Figure 8D**).

Further assessment of root vitality revealed that all three 35S::*AcHSFA2–2* lines exhibited significantly enhanced root vitality compared with WT. Qualitative analysis via 2, 3, 5−triphenyltetrazolium chloride (TTC) staining showed markedly deeper red coloration in roots of 35S::*AcHSFA2–2* plants relative to WT (**Figure 8E**). Quantitative measurements confirmed these observations: at 25 °C, root vitality in WT plants was 0.26 mg/g/h, whereas Line1, Line3, and Line4 displayed significantly increased values of 1.09, 0.76, and 0.88 mg/g/h, respectively (**Figure 8F**). AcHSFA2–2 over-expression enhanced root vitality by up to 4.13−fold compared with control.

**Figure 9:**
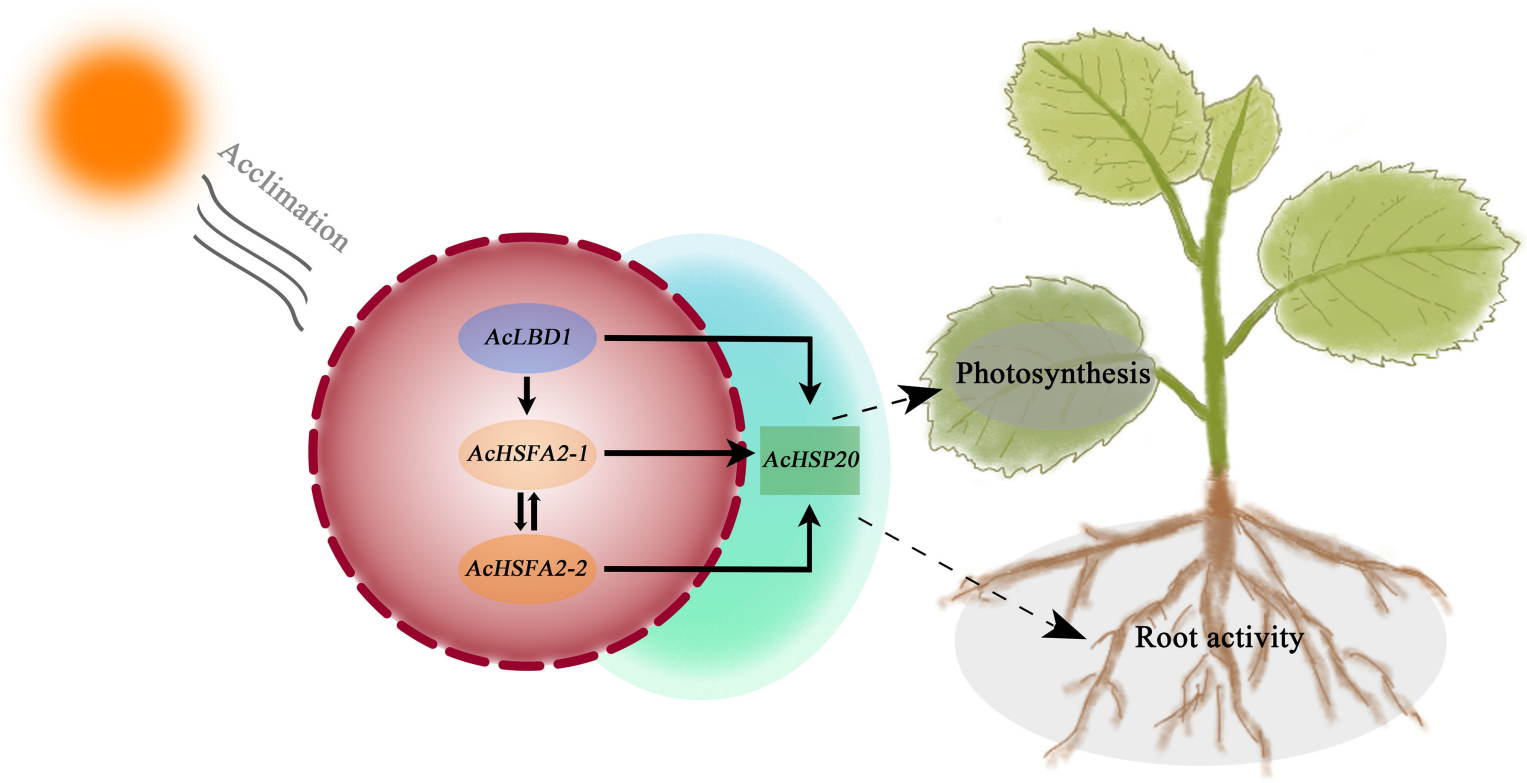
Schematic diagram illustrating how heat acclimation enhances root vitality and promotes post-heat-stress recovery via the mutual amplification cascade of AcLBD1 and AcHSFA2s in kiwifruit.

Furthermore, the transcript level of *AcHSFA2–2* also increased significantly during HA treatment (**Supplementary Figure 3**). Collectively, this study establishes a molecular network governing plant heat acclimation mediated by core genes including LBD, HSFA2, and HSP20. Following heat acclimation involving a transition from mild to extreme heat stress, *AcLBD1* is robustly induced and transcriptionally modulates the positive regulation loop formed by AcHSFA2–1 and AcHSFA2-2. This transcription factor module amplifies AcHSP20s expression, generating a mutual amplification cascade that ultimately drives rapid recovery of leaf net photosynthetic rate and root vitality (**Figure 9**).

## 4 Discussion

### 4.1 Responsive to temperature changes in the root ecosystem affect plant functionality

Root growth, development, and the underlying mechanisms governing their stress responses are considerably more complex and functionally critical throughout the life cycle of perennial plants.

Given the transient nature of atmospheric temperature fluctuations, young leaves serve as sensitive sensors for plants to perceive temperature stress. Consequently, the mechanisms by which the photosynthetic apparatus responds to temperature stress have been a central focus of most heat stress research (Kan et al., 2023; Zhao et al., 2025). Extensive evidence has demonstrated that heat stress impairs crop photosynthetic efficiency, leading to reduced yields; for instance, each 1 °C increase in global average temperature is associated with average yield declines of 6.0% for wheat, 3.2% for rice, 7.4% for maize, and 3.1% for soybean (Zhao et al., 2017). However, in evaluating physiological changes in kiwifruit leaves during heat stress, we observed a significant reduction in net photosynthetic rate under extreme heat stress, even though stomatal conductance, transpiration rate, and intercellular CO_2_ concentration remained unaltered (**Figure 2A**).

Previous studies have suggested that the detrimental effects of high ambient temperature on crop yield are also linked to its modulation of root system architecture (Veerappa et al., 2019; Lee et al., 2021). Roots exhibit a broad range of highly plastic physiological and morphological traits, enabling them to adjust their architecture and functionality in response to adverse environmental conditions. A robust overall plant phenotype inherently requires a well-established root system, which must therefore possess tolerance to multiple stressors. We thus incorporated root vitality as a key indicator to assess its association with plant thermotolerance.

The majority of existing studies have focused on root responses to moderate warming, which induce thermomorphogenic adaptations, rather than to acute heat stress. For example, elevated temperatures enhance lateral root length and number in *Arabidopsis* through the modulation of phytohormone signaling pathways (Wang et al., 2016; Martins et al., 2017; Feraru et al., 2018). However, there is a growing emphasis on deciphering the role of roots in plant adaptation to acute heat stress, driven primarily by widespread concerns regarding the improvement of crop productivity in agricultural systems (González-García et al., 2023). Plants respond to high temperatures by exhibiting largely tissue-specific patterns of gene expression; for instance, HY5 (ELONGATED HYPOCOTYL 5) regulates belowground root elongation, whereas PIF4 (PHYTOCHROME INTERACTING FACTOR 4) modulates aboveground tissue responses to heat (Lee et al., 2021). In the present study, we found that heat acclimation specifically induces *AcLBD1* expression, thereby enhancing root vitality, with *AcLBD1* transcript abundance displaying a tissue-specific pattern of high expression in roots (Actinidia eFP Browser). This finding further confirms that aboveground and belowground tissues employ distinct molecular mechanisms in their response to heat stress.

Similar to tolerance to other abiotic stresses, heat stress tolerance is a multigenic trait, and the candidate genes involved—particularly those regulating root vitality—remain poorly characterized. Our previous research identified differential expression of *AcMYB68* as a key factor contributing to the enhanced root vitality observed in waterlogging-tolerant kiwifruit genotypes (Chen et al., 2024). Roots are inherently difficult to evaluate in soil environments, and this technical challenge has been a major bottleneck limiting the improvement of stress tolerance in plant breeding programs (Calleja-Cabrera et al., 2020). In this study, we used tissue-cultured seedlings grown in a standardized, homogenized medium to eliminate potential confounding factors, including nutrient imbalances in the soil matrix and variations in rhizosphere microbial communities. TTC staining was employed to visually assess overall root growth, development, and vitality. However, several key questions remain to be addressed: how heat acclimation precisely modulates root vitality via *AcLBD1*, *AcHSFA2-2*, and potentially *AcHSFA2-1*; whether this modulation is associated with root thermomorphogenesis regulated by hormonal crosstalk; and whether it is linked to *AcMYB68*, which modulates root vitality in response to hypoxic stress.

In natural environments, increases in atmospheric temperature occur gradually over time; accordingly, we designed a heat acclimation regime that mimics this gradual transition from mild to extreme heat stress. Our finding that heat acclimation enhances root vitality holds significant practical implications for optimizing crop cultivation parameters in the rapidly expanding plant factory industry. For perennial plants, which encounter recurrent heat stress events throughout their life cycle—mirroring the heat acclimation treatment simulated in this study—an intriguing avenue for future research emerges: can perennial plants convert each annual heat stress event into thermomemory, thereby sustaining enhanced root vitality to support their perennial growth habit? This represents a highly promising direction for further investigation.

### 4.2 The response mode of *AcLBD1* to heat acclimation further expands the functional scope of LBD genes

LBD proteins, characterized by a conserved LATERAL ORGAN BOUNDARIES (LOB) domain, are key regulators of plant organ development, with a well-established role in lateral root initiation (Reviewed in Xu et al., 2016). As root-specific genetic regulators, LBD genes have been demonstrated to be essential for the initiation of seminal, shoot-borne, and lateral roots in maize (Taramino et al., 2007; Xu et al., 2015; Hochholdinger et al., 2018). In *Arabidopsis*, AtLBD11 and its downstream target *AtPLL18* (PECTATE LYASE-LIKE) promote radial root growth by modulating the pectin composition and mechanical properties of the primary cell wall (Ye et al., 2025). Furthermore, *KdLBD19* from *Kalanchoe* species can be exploited to improve crop transformation efficiency by enhancing the potential for plantlet regeneration (Meng et al., 2026).

Recent studies have extended the functional diversity of LBD genes beyond their canonical role in lateral organ boundary formation to encompass the regulation of stress responses. However, given the well-documented involvement of LBD genes in lateral root development, most research has focused on their mechanisms of response to soil-borne stresses, including salinity and nutrient limitation. For instance, the growth-promoting AtLBD11/ROS pathway is downregulated to fine-tune root growth in *Arabidopsis* under salt stress conditions (Dang et al., 2025). Similarly, *TaLBD27-3D* acts as a critical negative regulator of salt tolerance in wheat (Lu et al., 2025). In maize, *ZmLBD1* enhances tolerance to low phosphorus, NaCl, and drought stress, accompanied by improved root development (Yahaya et al., 2025). Collectively, these findings highlight a positive correlation between LBD gene expression, root growth, and abiotic stress resistance.

Consistent with these observations, our study demonstrated that heat acclimation significantly induces *AcLBD1* expression (**Figure 3B**) and concurrently enhances root vitality (**Figure 2C**). However, whether heat acclimation promotes lateral root formation as a means to enhance root vitality remains to be elucidated in future studies with extended experimental periods. Additionally, investigating whether LBD-mediated lateral root formation contributes to increased root vitality represents a promising direction for subsequent research.

Furthermore, LBD genes have been established as key components of the auxin signaling pathway that regulates root growth (Hochholdinger et al., 2018). Our findings revealed that AcLBD1 localizes to both the nucleus and the plasma membrane (**Figure 5A**), implying that AcLBD1 may also be involved in the perception and transduction of some signals during heat acclimation-induced enhancement of root vitality.

### 4.3 *AcHSFA2–1* and *AcHSFA2–2* mediate mutual regulation and exhibit distinct stress responsiveness

Plant heat shock factors (HSFs) are encoded by large gene families consisting of 18–52 members, which are categorized into three distinct classes (A, B, and C) (Nover et al., 1996). As integral components of complex signaling networks, HSFs govern plant responses not only to high-temperature stress but also to a range of other abiotic stresses, including cold, drought, and hypoxia (Andrási et al., 2021; Bakery et al., 2024). The kiwifruit transcriptome contains four *HSFA2* homologs that cluster within the same clade as *Arabidopsis AtHSFA2* (Ling et al., 2023; Shen et al., 2023). *AtHSFA2* has been well established as a critical regulator of heat acclimation in *Arabidopsis*; notably, it is the sole HSF required for the establishment of heat stress memory (Charng et al., 2007). Furthermore, the induction of *AtHSFA2* by heat acclimation is indispensable for both types of transcriptional heat stress memory characterized in *Arabidopsis* (Lämke et al., 2016; Yamaguchi et al., 2021). Our previous work confirmed that kiwifruit *AcHSFA2–1* responds to acute heat stress (Shen et al., 2023); however, whether other *AcHSFA2* members within the same clade share analogous functions and regulatory mechanisms remains to be fully elucidated.

Contemporary climate change has increased the frequency of combined environmental stresses—rather than isolated stress events—resulting in substantial global crop yield losses (Renziehausen et al., 2024). Beyond its role in heat stress responses, *AtHSFA2* is also implicated in the regulation of other abiotic stress responses in *Arabidopsis*. A particularly notable feature is its sequential regulatory role in mediating heat and hypoxic stress responses. Anoxia induces the expression of several heat shock proteins (HSPs), and mild heat pretreatment can acclimatize *Arabidopsis* seedlings to subsequent anoxic stress by promoting root growth (Banti et al., 2008). This acclimatory effect is abrogated, however, when the order of heat and anoxic treatments is reversed. This observation is attributed to the fact that anoxia strongly induces *AtHSFA2* and its target *HSP* genes in a heat-dependent manner (Banti et al., 2010). Collectively, these findings highlight a significant overlap between the molecular mechanisms underlying heat and anoxia tolerance, with HsfA2 functioning as a central mediator.

Inspired by the regulatory mechanism of *AtHSFA2* in *Arabidopsis* heat and anoxia responses, we analyzed the existing transcriptomic data from kiwifruit exposed to waterlogging stress which resulted in anoxia or hypoxia stress (Liu et al., 2022). Using the filtering criteria of |log_2_FC| ≥ 1, MaxFPKM ≥ 5, and *pvalue<* 0.05, we observed no significant alterations in *AcHSFA2–1* or *AcLBD1* expression under waterlogging stress. In contrast, *AcHSFA2–2* expression was markedly upregulated in response to waterlogging. We speculate that the activation of *AcHSFA2–1* by waterlogging may be dependent on concurrent or prior heat stress, analogous to the heat-dependent induction of *AtHSFA2* by anoxia in *Arabidopsis* (Banti et al., 2010). Notably, however, the induction of *AcHSFA2–2* by waterlogging in kiwifruit appears to be entirely independent of heat stress, representing a regulatory pattern distinct from that described in *Arabidopsis*. Our study further demonstrated that 35S::*AcHSFA2–2* kiwifruit plants exhibit enhanced root vitality and significantly improved thermotolerance (**Figure 8**); their responsiveness to waterlogging stress thus merits further investigation.

Additionally, this study clearly established a mutual transactivation regulatory relationship between *AcHSFA2–1* and *AcHSFA2-2* (**Figure 7**). Such inter-homolog regulatory crosstalk is absent in *Arabidopsis*, which possesses only a single *AtHSFA2* member. Although most foundational research on plant HSFs has been conducted in *Arabidopsis*, our understanding of HSF function is expanding to other plant species—including kiwifruit, a typical succulent, shallow-rooted perennial fruit tree. Elucidating the functions, interactions, and regulatory mechanisms of HSFs will facilitate the development of novel strategies for engineering crop genotypes with enhanced tolerance and adaptability to adverse environmental conditions.

## Data Availability

The raw data supporting the conclusions of this article will be made available by the authors, without undue reservation.

## Supplementary material

Supplementary Table 1 The primers list used in this study.
