# Supplementary material for: Heat acclimation induces AcLBD1 activated AcHSFA2s mutual amplification cascade for rapid recovery of root vitality and leaf photosynthesis in kiwifruit
Source: Front Plant Sci. 2026 May 5;17:1821100. doi: 10.3389/fpls.2026.1821100 (PMC13184819; doi:10.3389/fpls.2026.1821100)
Supplement: Supplementary file 2 [file DataSheet1.pdf]

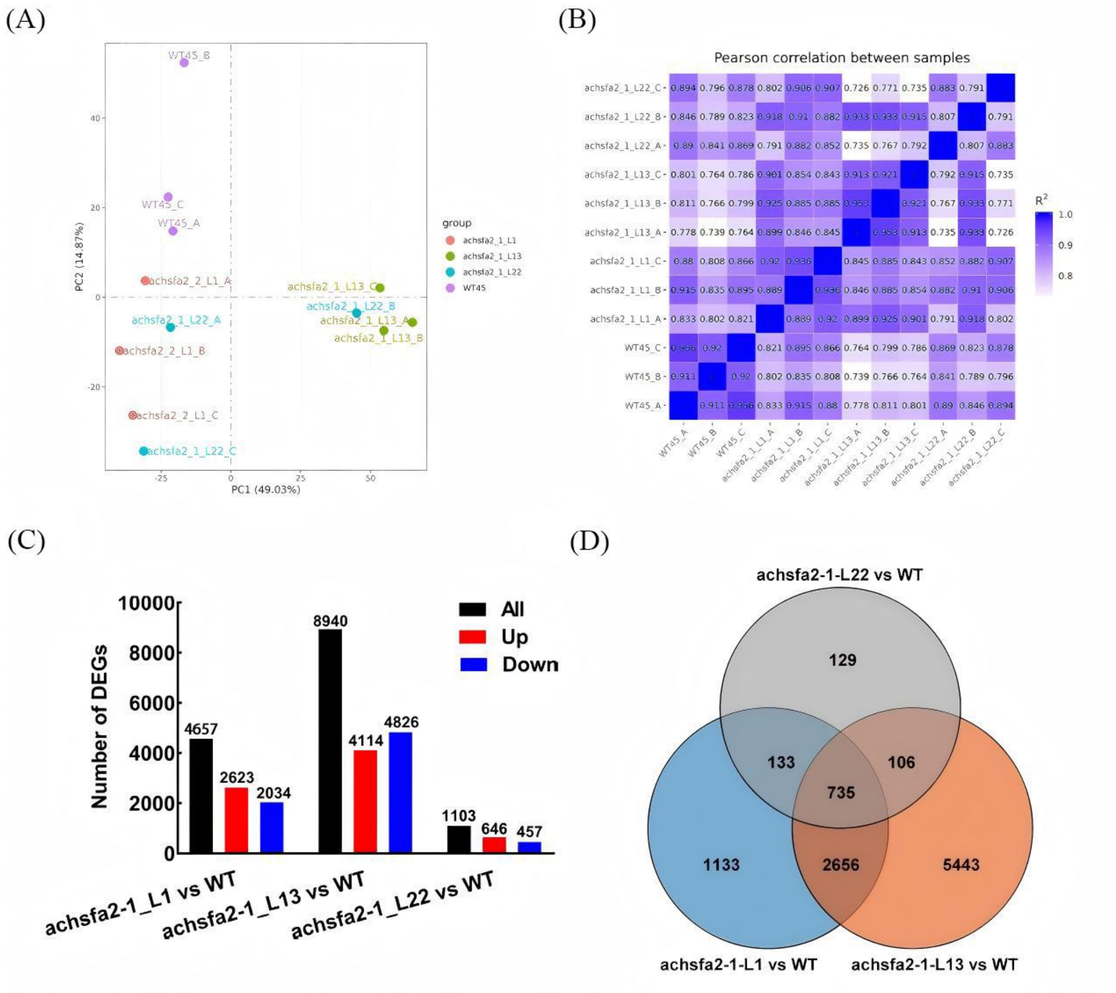

Figure S1 Transcriptome analysis of *achsfa2-1* loss-of-function mutants under heat stress treatment. (A, B) Principal component analysis (A) and Pearson correlation analysis (B) were performed to evaluate the stability of transcriptome data among three *achsfa2-1* mutant lines. WT45 represents wild-type plantlets subjected to heat stress treatment (45°C for 4 h). (C) Statistical analysis of the number of differentially expressed genes (DEGs). All indicates all DEGs, Up indicates up-regulated genes, and Down indicates down-regulated genes. (D) Venn diagram showing the similarities and differences in DEGs among different mutant lines.

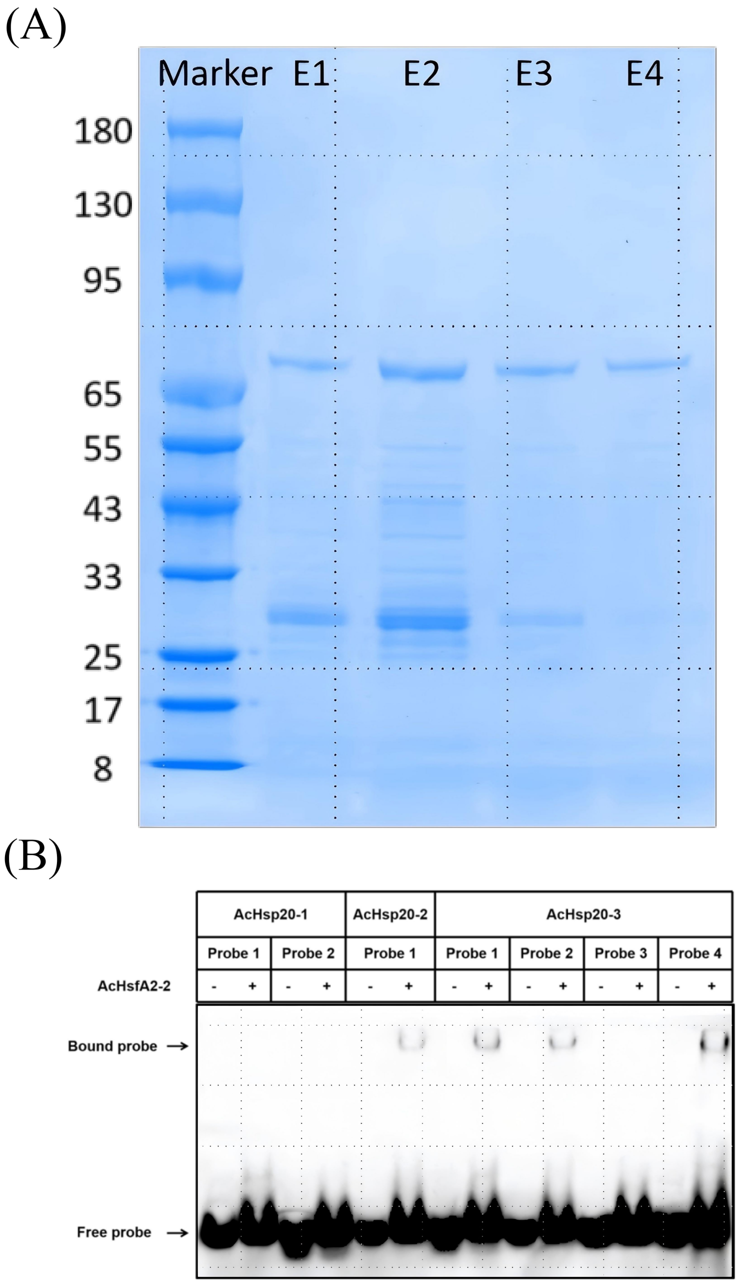

Figure S2 Verify the ability of AcHSFA2-2 to bind *AcHSP20-1/2/3* promoters based on EMSA. (A) Purification of AcHSF2-2 protein; (B) Binding effect of AcHSF2-2 with different probes located on promoters of *AcHSP20-1/2/3*. All probes except cold probes were labeled with 5'- and 3'-biotin. The cold probe does not contain a biotin label and is set up with three concentration gradients. '-' represent absence, '+' represent presence.

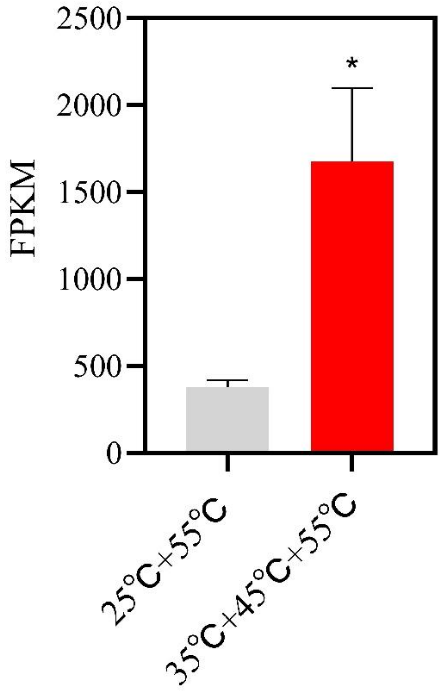

Figure S3 Expression of *AcHSFA2-2* in the heat acclimation transcriptome. 25°C + 55°C was used as the control group, and 35°C + 45°C + 55°C was used as the heat acclimation treatment group. Data were compared using FPKM (fragments per kilobase of exon per million fragments mapped) in the transcriptome. Error bars represent ±SE of three biological replicate samples in each experiment. Asterisks indicate significance levels: * P<0.05； ** P<0.01； *** P<0.001
