# Supplementary material for: Heat acclimation induces AcLBD1 activated AcHSFA2s mutual amplification cascade for rapid recovery of root vitality and leaf photosynthesis in kiwifruit
Source: Front Plant Sci. 2026 May 5;17:1821100. doi: 10.3389/fpls.2026.1821100 (PMC13184819; doi:10.3389/fpls.2026.1821100)
Supplement: Supplementary file 3 [file DataSheet2.pdf]

>Acc28668.1 [locus_tag=CEY00_Acc28668] [length=2061] [product=Heat shock factor protein] [manual_quality_score=2F] [status=full_length] [CDS=90-1298]
ATGGATGGTGTGACAGGGAAGGAGGAAGACATTACAGTGTGGCCGCAGCCGATGGAG
GGGCTACATGAGATGGGTCCGCCACCGTTTCTGACGAAGACCTTCGAAATGGTGGAGG
ACCCGTCGACTGATTCGGTTGTTTCGTGGAGCAGAGCTCGAAATAGCTTTGTCGTTAG
GGACTTGCACAAGTTCGCCACCACTCTGCTTCCGAGGTACTTCAAGCACTGCAATTTC
TCCAGCTTCGTTCGACAGCTCAATACCTATGGGTTCAAAAAAGTCGATCCAGACCAGT
GGGAATTCGCGAACGAAGGGTTTTTAGGTGGGCAGAAACATCTCTTGAAAACCATCAA
AAGGAGAAGAAATCTCTCCCACATCACGCCATTACATGGAGGATCGGGGGCTTGTGTG
GAGTTAGGCCAGTACGGAGTAGAAGAAGAAATCGAAAGGCTAACAATAGATCGCAGC
ATGTTAATGGCGGAAATCGTAAAAATCAACCAACAGCAGCAGAGCGCGAGGGACAAG
GTGATTGCAATGGAGGAGAGAGTGCAAAGCACCGAGAGGAAGCAACAACATATGATG
AACTTTCTTGCGAAGGCCTTAACTAGTCCTTCGTTTTTCAAGCAATTTATGGATAAGTAT
GTGGAGAGAAAAGAACAAAGAGGGGTCGAGATTGGACGGAAGAGGAGACTAACAAT
GAGCCCTAGTGTGGAGAGTTTTCAAGAACAAGAAGATTTGGTGATTATTGAATCAGAA
ATAGAGACATTGATTATGGATCCAAAAGCCGATCCTATTGTGACAACAAATGGTACCAA
CATGGATTCGGTTAGTGAGACTATTTGGGAGAAACTTTTTTGTGACGCCGGTGAGATTG
GACGGAAGAGGAGACTCGCAATGAGCCCTAGTGTGGAGAGTTTTCAAGAACAAGAAG
ATTTGGTGAATATTGAATCAGAAATTGAGACATTGATTATGGATCCAAAAGCCGATCCTA
TTGTGACAACTAATGGTACCGACATGGATTCGGTTAGTGAGACTATTTGGGAGAAACTT
TTTTGTGATGAAATTGTAGCCGGCGATGATGGCAATCAATCGGAATTTGACGTGGAGTT
TGAGGATTTGGCTGCGAAGACGACTCCGGATTGGGGAGAGGATTTACAAGACTTCGTT
GATCAGATGGAATTTCTCAGGTCGGACACCGATGACTTGTTTGGCTGA

*achsfa2-1*_L1
ATGGATGGTGTGACAGGGAAGGAGGAAGACATTACATGTGGCCGCAGCCGATGGAGG
GGCTACATGAGATGGGTCCGCCACCGTTTCTGACGAAGACCTTCGAAATGGTGGAGGA
CCCGTCGACTGATTCGGTTGTTTCGTGGAGCAGAGCTCGAAATAGCTTTGTCGTTAGG
GACTTGCACAAGTTCGCCACCACTCTGCTTCCGAGGTACTTCAAGCACTGCAATTTCTC
CAGCTTCGTTCGACAGCTCAATACCTATGGGTTCAAAAAAGTCGATCCAGACCAGTGG
GAATTCGCGAACGAAGGGTTTTTAGGTGGGCAGAAACATCTCTTGAAAACCATCAAAA
GGAGAAGAAATCTCTCCCACATCACGCCATTACATGGAGGATCGGGGGCTTGTGTGGA
GTTAGGCCAGTACGGAGTAGAAGAAGAAATCGAAAGGCTAACAATAGATCGCAGCATG
TTAATGGCGGAAATCGTAAAAATCAACCAACAGCAGCAGAGCGCGAGGGACAAGGTG
ATTGCAATGGAGGAGAGAGTGCAAAGCACCGAGAGGAAGCAACAACATATGATGAAC
TTTCTTGCGAAGGCCTTAACTAGTCCTTCGTTTTTCAAGCAATTTATGGATAAGTATGTG
GAGAGAAAAGAACAAAGAGGGGTCGAGATTGGACGGAAGAGGAGACTAACAATGAG
CCCTAGTGTGGAGAGTTTTCAAGAACAAGAAGATTTGGTGATTATTGAATCAGAAATA
GAGACATTGATTATGGATCCAAAAGCCGATCCTATTGTGACAACAAATGGTACCAACAT
GGATTCGGTTAGTGAGACTATTTGGGAGAAACTTTTTTGTGACGCCGGTGAGATTGGA
CGGAAGAGGAGACTCGCAATGAGCCCTAGTGTGGAGAGTTTTCAAGAACAAGAAGAT
TTGGTGAATATTGAATCAGAAATTGAGACATTGATTATGGATCCAAAAGCCGATCCTATT
GTGACAACTAATGGTACCGACATGGATTCGGTTAGTGAGACTATTTGGGAGAAACTTTT
TTGTGATGAAATTGTAGCCGGCGATGATGGCAATCAATCGGAATTTGACGTGGAGTTTG

AGGATTTGGCTGCGAAGACGACTCCGGATTGGGGAGAGGATTTACAAGACTTCGTTGA
TCAGATGGAATTTCTCAGGTCGGACACCGATGACTTGTTTGGCTGA

*achsfa2-1*_L13

ATGGATGGTGTGACAGGGAAGGAGGAAGAGTGTGGCCGCAGCCGATGGAGGGGCTAC
ATGAGATGGGTCCGCCACCGTTTCTGACGAAGACCTTCGAAATGGTGGAGGACCCGTC
GACTGATTCGGTTGTTTCGTGGAGCAGAGCTCGAAATAGCTTTGTCGTTAGGGACTTG
CACAAGTTCGCCACCACTCTGCTTCCGAGGTACTTCAAGCACTGCAATTTCTCCAGCTT
CGTTCGACAGCTCAATACCTATGGGTTCAAAAAAGTCGATCCAGACCAGTGGGAATTC
GCGAACGAAGGGTTTTTAGGTGGGCAGAAACATCTCTTGAAAACCATCAAAAGGAGA
AGAAATCTCTCCCACATCACGCCATTACATGGAGGATCGGGGGCTTGTGTGGAGTTAG
GCCAGTACGGAGTAGAAGAAGAAATCGAAAGGCTAACAATAGATCGCAGCATGTTAAT
GGCGGAAATCGTAAAAATCAACCAACAGCAGCAGAGCGCGAGGGACAAGGTGATTGC
AATGGAGGAGAGAGTGCAAAGCACCGAGAGGAAGCAACAACATATGATGAACTTTCT
TGCGAAGGCCTTAACTAGTCCTTCGTTTTTCAAGCAATTTATGGATAAGTATGTGGAGA
GAAAAGAACAAAGAGGGGTCGAGATTGGACGGAAGAGGAGACTAACAATGAGCCCT
AGTGTGGAGAGTTTTCAAGAACAAGAAGATTTGGTGATTATTGAATCAGAAATAGAGA
CATTGATTATGGATCCAAAAGCCGATCCTATTGTGACAACAAATGGTACCAACATGGAT
TCGGTTAGTGAGACTATTTGGGAGAAACTTTTTTGTGACGCCGGTGAGATTGGACGGA
AGAGGAGACTCGCAATGAGCCCTAGTGTGGAGAGTTTTCAAGAACAAGAAGATTTGG
TGAATATTGAATCAGAAATTGAGACATTGATTATGGATCCAAAAGCCGATCCTATTGTGA
CAACTAATGGTACCGACATGGATTCGGTTAGTGAGACTATTTGGGAGAAACTTTTTTGT
GATGAAATTGTAGCCGGCGATGATGGCAATCAATCGGAATTTGACGTGGAGTTTGAGG
ATTTGGCTGCGAAGACGACTCCGGATTGGGGAGAGGATTTACAAGACTTCGTTGATCA
GATGGAATTTCTCAGGTCGGACACCGATGACTTGTTTGGCTGA

*achsfa2-1*_L22

ATGGATGGTGTGACAGGGAAGGAGGAAGACAGTGTGGCCGCAGCCGATGGAGGGGCT
ACATGAGATGGGTCCGCCACCGTTTCTGACGAAGACCTTCGAAATGGTGGAGGACCCG
TCGACTGATTCGGTTGTTTCGTGGAGCAGAGCTCGAAATAGCTTTGTCGTTAGGGACTT
GCACAAGTTCGCCACCACTCTGCTTCCGAGGTACTTCAAGCACTGCAATTTCTCCAGC
TTCGTTCGACAGCTCAATACCTATGGGTTCAAAAAAGTCGATCCAGACCAGTGGGAAT
TCGCGAACGAAGGGTTTTTAGGTGGGCAGAAACATCTCTTGAAAACCATCAAAAGGA
GAAGAAATCTCTCCCACATCACGCCATTACATGGAGGATCGGGGGCTTGTGTGGAGTT
AGGCCAGTACGGAGTAGAAGAAGAAATCGAAAGGCTAACAATAGATCGCAGCATGTTA
ATGGCGGAAATCGTAAAAATCAACCAACAGCAGCAGAGCGCGAGGGACAAGGTGATT
GCAATGGAGGAGAGAGTGCAAAGCACCGAGAGGAAGCAACAACATATGATGAACTTT
CTTGCGAAGGCCTTAACTAGTCCTTCGTTTTTCAAGCAATTTATGGATAAGTATGTGGA
GAGAAAAGAACAAAGAGGGGTCGAGATTGGACGGAAGAGGAGACTAACAATGAGCC
CTAGTGTGGAGAGTTTTCAAGAACAAGAAGATTTGGTGATTATTGAATCAGAAATAGA
GACATTGATTATGGATCCAAAAGCCGATCCTATTGTGACAACAAATGGTACCAACATGG
ATTCGGTTAGTGAGACTATTTGGGAGAAACTTTTTTGTGACGCCGGTGAGATTGGACG
GAAGAGGAGACTCGCAATGAGCCCTAGTGTGGAGAGTTTTCAAGAACAAGAAGATTT
GGTGAATATTGAATCAGAAATTGAGACATTGATTATGGATCCAAAAGCCGATCCTATTGT
GACAACTAATGGTACCGACATGGATTCGGTTAGTGAGACTATTTGGGAGAAACTTTTTT
GTGATGAAATTGTAGCCGGCGATGATGGCAATCAATCGGAATTTGACGTGGAGTTTGA

GGATTTGGCTGCGAAGACGACTCCGGATTGGGGAGAGGATTTACAAGACTTCGTTGAT
CAGATGGAATTTCTCAGGTCGGACACCGATGACTTGTTTGGCTGA
